# The ‘Drug Bag’ method: lessons from anthropological studies of antibiotic use in Africa and South-East Asia

**DOI:** 10.1080/16549716.2019.1639388

**Published:** 2019-07-24

**Authors:** Justin Dixon, Eleanor MacPherson, Salome Manyau, Susan Nayiga, Yuzana Khine Zaw, Miriam Kayendeke, Christine Nabirye, Laurie Denyer Willis, Coll de Lima Hutchison, Clare I. R. Chandler

**Affiliations:** a Department of Global Health and Development, London School of Hygiene & Tropical Medicine, London, UK; b Liverpool School of Tropical Medicine, Malawi-Liverpool Wellcome Trust, Blantyre, Malawi; c Biomedical Research and Training Institute, Harare, Zimbabwe; d Infectious Diseases Research Collaboration, Kampala, Uganda

**Keywords:** Antimicrobial Resistance, Antibiotic use, antimicrobial resistance, household surveys, pile sorting

## Abstract

Understanding the prevalence and types of antibiotics used in a given human and/or animal population is important for informing stewardship strategies. Methods used to capture such data often rely on verbal elicitation of reported use that tend to assume shared medical terminology. Studies have shown the category ‘antibiotic’ does not translate well linguistically or conceptually, which limits the accuracy of these reports. This article presents a ‘Drug Bag’ method to study antibiotic use (ABU) in households and on farms, which involves using physical samples of all the antibiotics available within a given study site. We present the conceptual underpinnings of the method, and our experiences of using this method to produce data about antibiotic recognition, use and accessibility in the context of anthropological research in Africa and South-East Asia. We illustrate the kinds of qualitative and quantitative data the method can produce, comparing and contrasting our experiences in different settings. The Drug Bag method can produce accurate antibiotic use data as well as provide a talking point for participants to discuss antibiotic experiences. We propose it can help improve our understanding of antibiotic use in peoples’ everyday lives across different contexts, and our reflections add to a growing conversation around methods to study ABU beyond prescriber settings, where data gaps are currently substantial.

## Background

In response to rising concerns around antimicrobial resistance (AMR), the WHO’s [1] Global Action Plan has called for improved understanding and surveillance of antibiotic use (ABU) in human and animal populations. In low- and middle-income countries (LMICs), where much of the recent growth in ABU has occurred, the most cited methods and metrics to shed light on ABU come from macro-level consumption data from sales and imports [2,3] and from prescriber settings, especially hospitals [4]. However, to be able to ascertain the risks of ABU for AMR in different scenarios, and therefore to guide investment into efforts to curb AMR through reducing ABU, fine-tuned methods are needed to understand how much and which antibiotics are being used and by whom outside formal healthcare settings. Currently there is no agreed protocol for collecting such data at the local level, which is particularly important in countries with active informal healthcare markets.

In a recent review of ABU data collection methods, Queenan, Chandler and Goodman [5] found that most household surveys relied upon respondent recall and inventories of antibiotics kept at home – sometimes aided by the use of show-cards – while farm surveys utilised various combinations of recall, treatment logs and packaging-bin searches. Among the challenges faced by surveys is that recall is notoriously unreliable [6] and inventories are restricted by what is actually kept at home. In addition, surveys usually take place at a single time point, affecting reliability of data collected due to respondents having limited time to get to know the reasons for the research [7,8] as well as the ability for snapshot data to reflect wider trends and issues, for example in illness episodes [9]. For ABU surveys, these challenges are compounded by the fact that ‘antibiotic’ is a broad and complex category that includes numerous medicines and classes with long names and in some scenarios substantial brand variation. Moreover, it is often assumed in surveys that respondents recognise antibiotics according to biomedical concepts and categories [10] when in fact they may not use notions such as ‘bacteria’, ‘antibiotic’ or ‘infectious disease’ at all [11].Together, these challenges significantly limit the reliability of data that ask verbally about ABU in a one-off interaction.

Nonetheless, an understanding of ABU at the granular level is key for well-informed interventions and to avoid making assumptions about the prevalence and nature of ABU. Ethnographic research, including immersive and observation techniques, is ideal for understanding why and how medicines are used, and for capturing local categories of medicines and the contexts of their use [12,13]. For example, Muelenbroek found during fieldwork in Burkina Faso that the meaning of medicines was symbolically tied to objects and places in the body, making illness episodes tangible and therefore treatable [12]. Survey methods can complement ethnographic approaches – not for the purpose of capturing ‘attitudes’, ‘beliefs’ or ‘culture’ [7,14,15] – but to understand the extent to which populations are using different antibiotics.

This article presents findings and experiences from using what we call the ‘Drug Bag’ method, which we have been using as part of household and farm surveys in Africa and South-East Asia as a precursor to or alongside ethnographic fieldwork. In the following, we firstly discuss the methodological and conceptual underpinnings of the method, before describing how we have operationalised it using an electronic data capture system. We then draw on preliminary findings from projects in Zimbabwe, Malawi, Uganda and Myanmar to show the various kinds of qualitative and quantitative data that the method can produce. We finally reflect upon the strengths and limitations of the method, and how these can inform the conduct of larger ABU surveys and more generally contribute to the growing conversation around ABU data collection methods beyond prescriber settings.

## Methodology

### Approach

The Drug Bag method is a modified version of the established anthropological method of ‘pile sorting’ and draws on increasing concern to engage in interview methods that are not solely cerebral but that use physical materials to stimulate a deeper conversation between interviewer and interviewee [16,17]. Pile sorting was originally designed for mapping ‘cultural domains’ and involves asking people to sort things into piles based on similar attributes [13]. Pile sorting has had wide applications within and beyond anthropology, including in survey research [18]. In medical anthropology, it has been used to study a range of themes such as illnesses, medicines, norms, values and perceptions of risk [19–22]. What makes pile sorting useful for studying ABU is its use of visual and/or material cues to assemble different ‘piles’ based on predefined criteria. Our basic idea was to disentangle pile sorting from the study of cultural domains and repurpose its material and visual nature to the more modest task of finding out, without assuming that people deploy biomedical terms (e.g. ‘antibiotic’), which antibiotics people recognise, use and can(not) access. This engages the materiality of medicines [12] to establish common referents with our respondents that would allow us to build a bridge between ‘local’ and ‘global’ categories.

### Implementation

We developed and piloted the Drug Bag method within our ongoing ethnographic studies in Harare in Zimbabwe, Chikwawa District in Malawi, Yangon in Myanmar, and Kampala and Tororo in Uganda. We conducted one study of ABU in the animal sector, involving a rural-urban comparison in Wakiso District and Tororo, Uganda (see Table 1). Our research teams carrying out the drug bag studies comprised of social science research degree students and experienced research assistants. Our objectives for the drug bag exercises are presented in Box 1.

**Box 1.** Drug Bag Research Objectives

To establish how many, and which, different antibiotics were recognisable by members of households and/or farmers in a given study context To capture which antibiotics household members/farmers said they used, and the approximate frequency of use To capture the symptoms and illnesses that frequently-used antibiotics were used to treat. To capture which antibiotics were easier and harder to access

**Table 1.** Drug Bag participants

Study Site (Region, Country)	Human/animal sector	Sample Size	Mean Household Size	Sex of main respondent (% female)	Mean age and range of main respondent
Harare, Zimbabwe	Human	100	6	96	43 (18–81)
Chikwawa, Malawi	Human	101	4.5	90	27 (17–71)
Yangon, Myanmar	Human	50	5	92	47 (21–83)
Kampala, Uganda	Human	174	-	79	-
Tororo and Wakiso, Uganda	Animal	115	-	52	18–87

**Figure 1.**
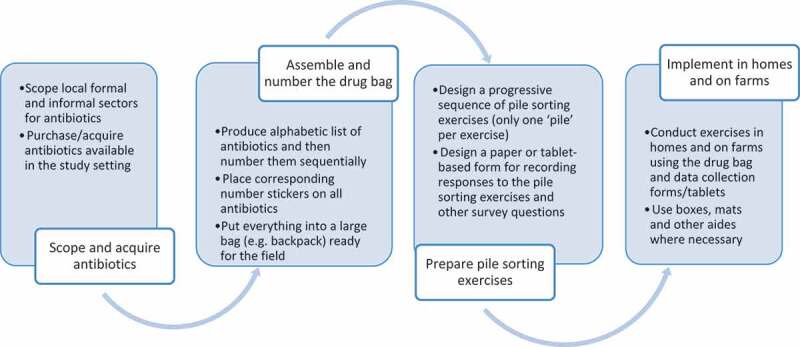
Steps taken to operationalise drug bags

Figure 1 shows the various steps that we took while assembling and operationalising our drug bags in our different settings. In all study sites, we started by visiting all spaces where antibiotics could be acquired, including local clinics and hospitals, retail pharmacies, vets (for the animal ABU survey), as well as iterant drug sellers and market vendors. We generally started with providers nearest to residential areas/farms, before moving further afield until we had exhausted as many options as reasonably possible. We then bought as many different types of antibiotics as we could, making sure to include differences in ingredients, appearance, brand name and mode of administration to optimise recognition. In some surveys, we did include eye drops, ear drops and topical creams. However, most emphasis on ABU data collection globally is on oral and injectable antibiotics [2], and thus we restrict our data in this article to these modes of administration. In most instances, the generic name was included on the packaging, but where it was not a label was added and/or respondents were informed of the names as they examined the drugs. In our African settings, we found between 40–60 antibiotics; in Myanmar, we found well over 100, which reflects the larger market for antibiotics in South-East Asia [23]. The antibiotics were listed alphabetically, and each was assigned a number from 1-n. This number was recorded on the physical copy of each antibiotic via a sticker, such that we ended up with a one-to-one correspondence between the list and the drug bag.

We then designed a series of pile sorting exercises involving the drug bags that were included within household and farm surveys in our study settings (surveys also included questions about common illnesses, medicines and health seeking to contextualise antibiotic use). Given the anthropological orientation of our research, we did not design these surveys as self-standing studies of ABU but rather a precursor to or alongside longer-term ethnographic fieldwork. As a result, it was not necessary in our work to calculate statistically-defined sample sizes and generate widely representative data, although the lessons we learned could and indeed are being fed into the design and conduct of larger surveys, including an ongoing survey in Chikwawa, Malawi. Most surveys involved purposive sampling of households or farms, with the aim of ‘getting to know’ our study populations and sub-groups within them. The exercises were pitched at the level of the farm and/or household. Wanting to produce a rich, holistic picture of ABU, there was usually a primary respondent (e.g. household head or farm owner), but we actively encouraged other present household/farm members to join in with the exercises.

**Figure 2.**
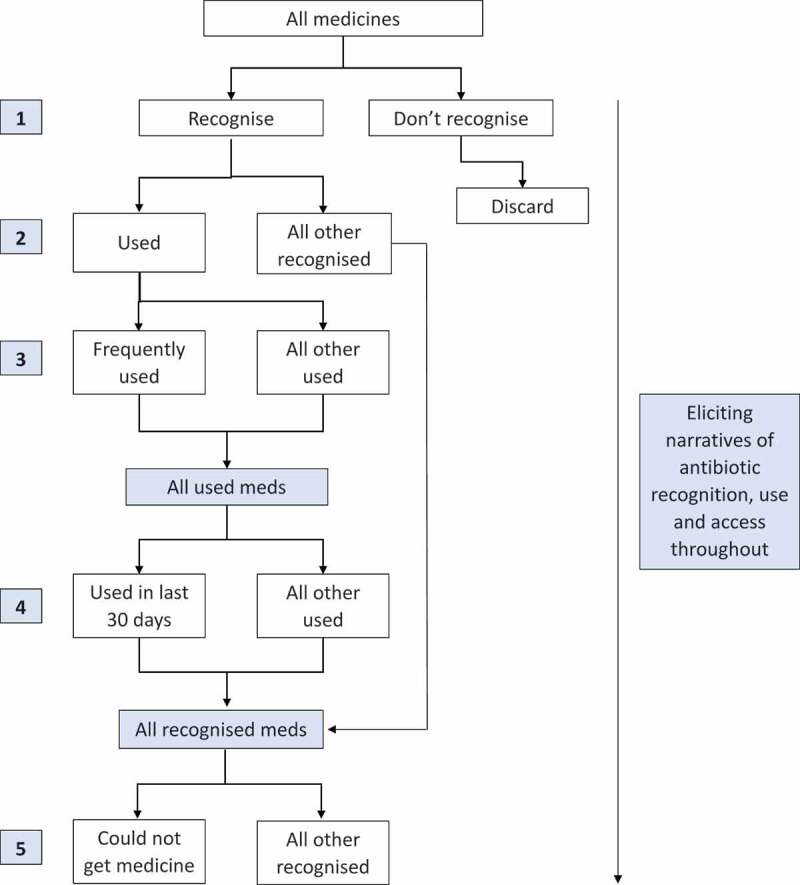
Flow diagram showing the progression of pile sorting exercises

In order to keep the process as simple and reliable as possible, the pile sorting exercises were designed to be sequential, cumulative and only ever involve one ‘pile’ being created each time. The task of the first exercise was to identify which of the large collection medicines in the bag that respondents recognised and which they did not. This normally enabled us to exclude the vast majority of antibiotics, resulting in a smaller, more manageable collection of antibiotics to be used for the remaining exercises. While the ensuing exercises could have been taken in a number of directions, our objectives (Box 1) led us to ask respondents to pick out which ones they had used before; which they used frequently in their household when someone was sick; which they had used in the last 30 days; and which they had at times been prescribed or wanted but struggled to access (Figure 2). The ‘piles’ were recorded on tablets using Open Data Kit (ODK) (accessible at www.opendatakit.org) and analysed quantitatively using R (accessible at https://www.r-project.org). However, the data could also have been collected on paper forms and entered subsequently into a computer database.

**Figure 3.**
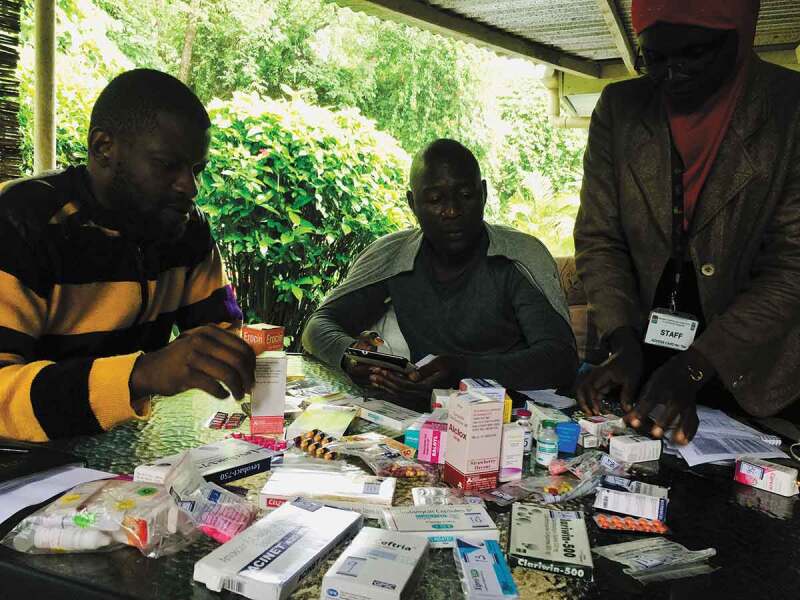
Field team in Malawi practicing the pile sorting exercises

In order to capture the ‘hows’, ‘whys’ and ‘whens’ of ABU – and to thereby qualify, substantiate or challenge quantitative data – we elicited respondents’ narratives throughout. They were encouraged to narrate why medicines were placed in certain piles, which illnesses ‘frequently used’ medicines were used to treat, and any stories that respondents had to tell about using (or being unable to access) the medicines. To capture these narratives, we either audio recorded the pile sorting process or took detailed field notes. Thematic analysis was then conducted on the data using NVivo 11. In Malawi, where narrative data were not captured, we found that the exercises took between 15–40 minutes (Figure 3). With the narrative component included, then depending on various factors including how talkative people were, how much time they had available, and any distractions, the exercises could take anything from 30 minutes to over two hours. A copy of one of the versions of the medicines survey can be found here.

## Findings of the Drug Bag method in action

### Participants

We used the Drug Bag Method with five samples of households and farms across our settings, as described in Table 1. These numbers inform our analysis of the utility of the method at the time of writing.

### Did the Drug Bag method produce useful data on antibiotic recognition?

**Figure 4.**
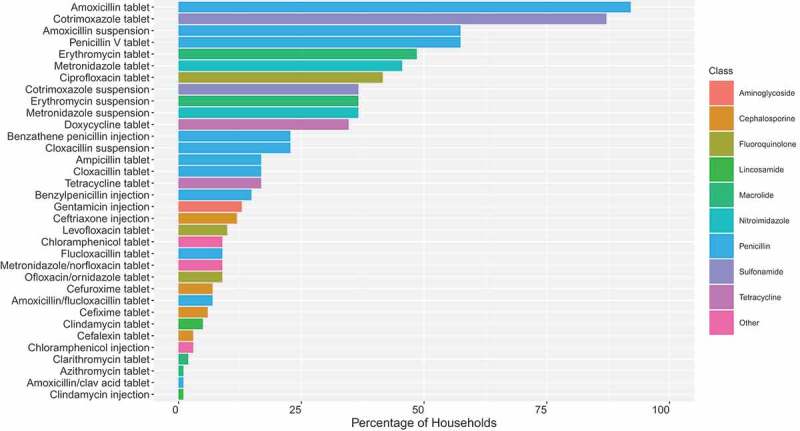
Percentage (%) of respondents that recognised antibiotics available in Chikwawa District, Malawi (n = 101)

During the design phase of our research, we queried whether people would actually recognise many antibiotics, even when presented with physical examples (objective 1). We found that at our African sites, respondents recognised many of the antibiotics we showed them, especially oral antibiotics (tablets, capsules and suspensions). The antibiotics that most commonly featured in the ‘recognised’ pile were penicillins (especially amoxicillin), doxycycline, cotrimoxazole, metronidazole, ciprofloxacin and erythromycin. These antibiotics were generally those that were available in primary healthcare facilities, funded through donor programmes and/or available in the informal sector. To exemplify, Figure 4 shows our quantitative recognition data from Chikwawa District, Malawi. Here amoxicillin and cotrimoxazole tablets stand out in particular because, as we found, these are often the only two stocked in primary clinics. Across all of our sites, people tended to be less familiar with injectable forms of antibiotic and those more usually prescribed in hospital settings (e.g. ceftriaxone). Nonetheless, we encountered many people who recognised antibiotics that they or others had been prescribed as inpatients. In the context of farming in Uganda, meanwhile, the most widely recognised antibiotics for poultry were tetracyclines and sulfonamides, mostly in powder form added to poultry drinking water, and salinomycine included in purchased feeds. The types of antibiotics remained the same for pigs, but in their case, injectables were much more common.

*Includes 34 antibiotics classified by generic name and mode of administration (and reclassified retrospectively by class). Tablets and capsules are both labelled ‘tablet’.

In Myanmar, most respondents were familiar with oral forms of amoxicillin and ampicillin. However, beyond these, levels of recognition amongst our respondents seemed low, and our researchers reported much ‘guessing’ by respondents, who often felt like they were being tested. Low levels of recognition might be surprising given the large informal health sector in Myanmar, in which many antibiotics can be bought without a prescription. However, as we found while scoping for antibiotics (and as is widely documented in South-East Asia [23]), drug shops often do not give drugs in their original packaging but rather take them out and provide a certain number of doses (often alongside other medicines) in small plastic bags. With so many antibiotics looking the same or very similar, this meant that our respondents were often unable to pick out even those antibiotics to which they might be regularly exposed.

**Figure 5.**
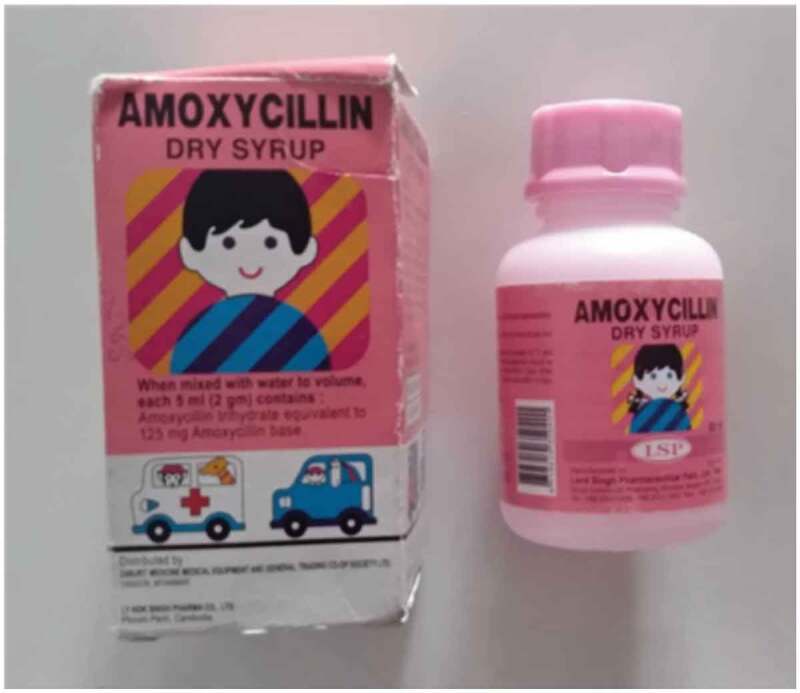
The ‘jeep-car medicine’ in Yangon, Myanmar

Although establishing recognition of medicines in Myanmar was challenging, in all sites we paid close attention to the way that respondents spoke with us and each other and were able to ascertain not only which antibiotics they recognised but *how* they recognised them. In Zimbabwe, for instance, generic names were shorthanded to ‘cipro’ (ciprofloxacin), ‘doxy’ (doxycycline), ‘amoxyl’ (amoxicillin) and ‘cotri’ (cotrimoxazole). But also, we saw branding and packaging influencing local categories. For example, in Myanmar, where there was considerable brand variation, one of the antibiotics, an amoxicillin suspension often given to children in the area, was referred to locally as the ‘jeep-car medicine’ (Figure 5), because of the picture on the box. Identifying local categories and terminologies was an important starting point for exploring people’s relationships with medicines and how knowledge is communicated within and across formal and informal, professional and popular sectors. It allows researchers to move beyond technical terms and jargons that can impede more open discussions of ABU.

### Did the Drug Bag method produce useful ABU data?

**Figure 6.**
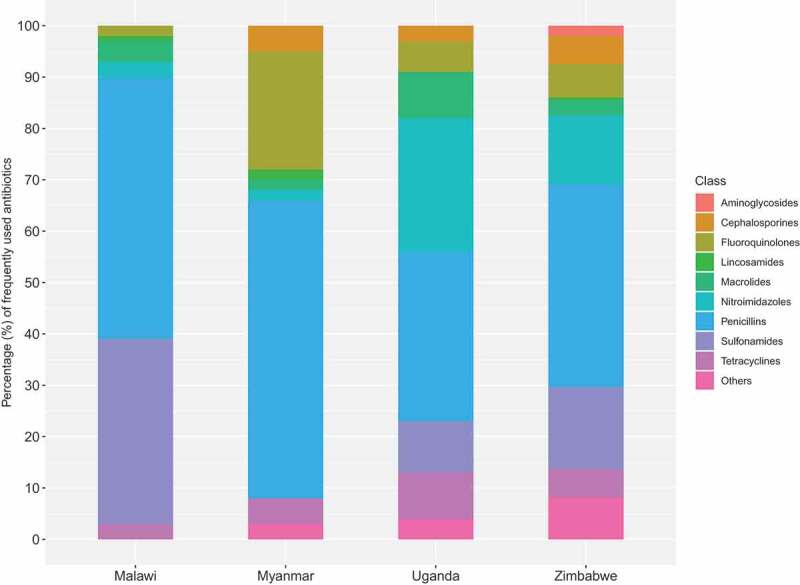
Proportional reported use (%) of frequently-used antibiotics by class among households in Malawi (n = 101), Myanmar (n = 50), Uganda (n = 174) and Zimbabwe (n = 100)

Upon the foundations of our recognition data, we began to identify patterns of household and farm antibiotic use within and between our research sites, as well as differences between them. One of the most important objectives for orienting further fieldwork was to identify those antibiotics that our respondents were using frequently and that were making up the bulk of ABU (objective 2). As we found, antibiotics placed in the ‘frequently used’ pile were also the most likely to have been used within the 30 days prior to the survey. Among our respondents in Harare, Zimbabwe, the most ‘frequently used’ antibiotics were amoxicillin, cotrimoxazole, doxycycline, metronidazole, ciprofloxacin and erythromycin (the same as the most recognised antibiotics), and on average this pile contained 4.3 antibiotics. By contrast, in Chikwawa, the pile contained on average only 1.2 antibiotics, usually amoxicillin and/or cotrimoxazole. This was unsurprising in this particular setting given the extremely limited availability of antibiotics in the area. Uganda fell just between Zimbabwe and Malawi, with 2.8 antibiotics in the ‘frequently used’ pile, and metronidazole making up 26% of ABU, a markedly different pattern than in our other sites. In Myanmar, the pile tended to be similarly small with varying composition, but this is likely related to the difficulties of establishing recognition. Figure 6 shows the proportional consumption of frequently-used antibiotics by class in Malawi, Myanmar, Uganda and Zimbabwe.

Respondents’ narratives provided a richer understanding of the illnesses that particular antibiotics were used to treat (Objective 3), as well as how knowledge about antibiotic use was acquired and shared. Picking up antibiotics and conversing in animated tones among one another, we found, for instance, that respondents in Uganda used metronidazole to treat both a range of stomach problems and chronic pain; that in Zimbabwe, metronidazole was used along with ciprofloxacin or doxycycline to treat STIs; that in Malawi, cotrimoxazole given to HIV-positive individuals was often shared with other household members when they were sick; and that tetracycline powders were used by Ugandan farmers to care for poultry with recurrent gastrointestinal and respiratory conditions. ABU often followed attendance at a clinic, hospital, or vet, sometimes involving a prescription taken to a retail pharmacy. But knowledge of antibiotic use also filtered from and circulated beyond the formal sector, as old prescriptions and past experiences were used to inform ABU or to guide purchasing drugs through the informal sector. It was during these lively discussions that we began to appreciate how effective the Drug Bag method was for provoking memories and experiences of ABU and for helping us to understand how, why and when different antibiotics were used in the context of people’s everyday lives and livelihoods.

### Did the Drug Bag method produce useful data on antibiotic accessibility?

Given the precarious circumstances in which many of the households and farmers across our study sites lived and worked, it is impossible to understand patterns of ABU without considering the accessibility of different antibiotics (Objective 4). When respondents were asked to pick out those antibiotics they had ever needed before but been unable to access, we found that some of the more expensive antibiotics generally found in the hospital sector were placed in this pile. For instance, in Harare, Zimbabwe, more households reported having needed and being unable to access vancomycin – a drug that costs upwards of $30USD per day for hospital inpatients – than had actually ever used the drug. But perhaps more importantly, even access to basic antibiotics appeared to be highly temperamental. For example, amoxicillin, the most widely-used antibiotic at the primary care level globally, was also the most commonly placed in the ‘needed but could not access’ pile. Our respondents further substantiated this by drawing attention to long clinic queues, long distances needed to travel to reach clinics, frequent stockouts, user fees and extortionate pharmacy prices as common reasons for non-access. With numerous socio-economic factors undermining people’s ability to access medicines through the formal sector, for many, the informal sector was often the only available option to them.

The main challenge of establishing the accessibility of different antibiotics using our particular method was that many of the drugs that we suspected would be harder to access – e.g. later-generation cephalosporins or carbapenems – were sparsely recognised by our respondents or not even included in the drug bags at all. Speaking in terms of the WHO’s new Access, Watch and Reserve (AWaRe) categories, many of the more expensive antibiotics belong to the ‘Watch’ or ‘Reserve’ groups. ‘Key Access’ antibiotics are those which should be ‘widely available, affordable and quality-assured’; ‘Watch Group’ antibiotics are those with a higher resistance potential and to be prioritized as key targets of stewardship interventions; and ‘Reserve Group’ antibiotics are ‘last resort options’ [24, 8–9]. While the majority of the antibiotics in our drug bags belonged to the ‘Access’ group, the bulk of the cost of putting together the bags went into buying ‘Watch’ group antibiotics, and we could only find two ‘Reserve’ list antibiotics at all, both of which were in Myanmar. Overall, we got a better picture of antibiotic availability through the process of assembling the bags, hearing respondents talk about the inequities of the health system and noting that the overwhelming majority of ‘frequently-used’ drugs were on the ‘Access’ list. Even in Myanmar, where antibiotics were cheaper and respondents rarely reported being unable to get antibiotics when needed, we realised that this was not equivalent to access to *care*. Indeed, it seemed that accessing good quality care was, if anything, more challenging than in Africa.

### What unintended effects did the Drug Bag method have?

Walking into people’s houses and farms with a large bag of drugs that people were often either unfamiliar with or were unable to access had unintended consequences. Sometimes, respondents would ask to keep the drugs that we had brought with us; people were sick, and we had medicines that might help to make them better. Moreover, the drug bags brought with them new ideas and knowledge that was put to use, most evidently in our Ugandan farm survey. For example, one respondent said that she gained new knowledge during the survey which she applied to her chicken farming:

When I saw her drugs … I didn’t know them. After, I went to the vet and remembered the colour and bought that drug. I researched it, tried out the medicine on my chickens. They are now well. Even my neighbours now they are wanting to use this medicine.

Another respondent’s cows were doing so well that after that her neighbours stole them. As it transpired, respondents were taking their own photos and notes of unfamiliar medicines and buying them from drug shops. This served as a stark reminder that, by bringing people into contact with new medicines, we were not simply ‘neutral’ observers but became active participants, understood as ‘experts’, shaping antibiotic knowledge and use. We return to the ethical and methodological implications of these effects below.

## Discussion

Understanding the prevalence and types of antibiotics used in a given population is important for informing interventions that attempt to control ABU. In low-income settings where digital prescribing records capture the minority of antibiotic prescriptions or purchases, community-level data collection is required to evaluate the local contours of ABU. This paper presents methodological developments in the assessment of antibiotic use through the ‘Drug Bag’ approach. An adaptation of ‘pile sorting’ [13], the limitations of spoken surveys that rely on abstract concepts of antibiotics are circumvented through the material presentation of antibiotics that are then sorted into piles according to the research questions. Our accounts of piloting this approach in Zimbabwe, Malawi, Uganda and Myanmar showcase the kinds of data the method can produce as well as highlight its strengths and limitations.

### Logistics

Operationalising the drug bags took 2–5 weeks for our field teams to complete, including scoping, purchasing, and assembling the bags (Figure 1). Oral antibiotics were generally very cheap, but some injectable medicines cost up to USD30. The total cost of the drug bags ranged between USD70 (Malawi) and USD350 (farm drug bag, Uganda). While this was time and resource intensive, we learned much about local pharmaceutical markets along the way, which was valuable data in the context of our broader project objectives. Indeed, aside from the knowledge gained, we established relationships with local sellers, several of whom since became enrolled in ethnography.

In terms of implementation, using the Drug Bag Method was not as time consuming as we had first expected, because the initially large number of antibiotics tended to be whittled down to a manageable few following the first recognition exercise. It was also social and often fun for respondents, resulting in high rates of interest in participating. Capturing qualitative data alongside the exercises took the longest and could take anywhere between 30 mins and 2 hours. In Malawi, where qualitative data were not captured, we found this took 15–40 minutes. Future studies could expand or adapt the pile sorting exercises to answer particular research questions, and it will fall to careful design and piloting work to determine what can be accomplished within the time constraints of the research.

### Assessing data quality against objectives

In terms of the utility of the Drug Bag method in meeting our objectives, establishing recognition was most successful at our African sites, where there was a relatively small and stable range of antibiotics available. Recognition was highest among oral antibiotics that are most commonly available in public clinics, from vets and in the informal sector. In Myanmar, although we were able to shed light on the ways that people categorise and speak about particular antibiotics, establishing patterns of recognition was more challenging. As suggested above, this was likely related to the large informal markets for antibiotics and the way that medicines are bought and sold [11,23]. In Myanmar, if the drug bags made one thing clear, it was that perhaps we had started in the wrong place; if we wanted to understand people’s relationships with medicines, we had to understand their relationship with providers of antibiotics. Moreover, social scientists in Myanmar [25,26] have noted that the country’s long history of authoritarian rule has left many people highly suspicious and fearful of unknown outsiders probing into their lives. This is a political context that international health research cannot easily escape: it made it very difficult to establish trust and almost certainly affected the way that people responded to the drug bags.

Where we arguably had the most success using the Drug Bag method was in establishing which antibiotics people were ‘frequently using’ at the household and farm level (objective 2) and what those antibiotics were used to treat (objective 3). The most frequently-used antibiotics tended to be the most recognised and, particularly in our African sites, we were encouraged by the confidence with which people picked out and spoke about how, why and when such antibiotics were used. While self-reported ‘frequent use’ by no means captures the full extent of ABU, such categories offer a window into the local contours of ABU beyond prescriber settings where data are currently fewest. In order to shed further light on how and why people use antibiotics the way they do, we also used the drug bags to assess the accessibility of antibiotics in different settings (objective 4). Our data on reported accessibility was constrained by what people were able to recognise within the drug bags, and thus we had limited success with meeting objective 4. Nevertheless, we were able to learn more about antibiotic access and availability from assessing what kinds of drugs people were (frequently) using, where they got them from, and hearing about the difficulties that people had accessing healthcare in contexts of often-extreme poverty [27]. In sum, even if reliable use data is restricted to a select few widely-recognised, frequently-used antibiotics, understanding these medicines and the contexts of their use is important for designing targeted and context-specific interventions to address AMR.

### Ethical implications of the Drug Bags

Working with physical samples of antibiotics – material objects with significance and value – raised a number of ethical issues. Studies have shown that field workers often play an under-acknowledged ethically-charged role mediating between the interests and expectations of researchers and study communities [28,29]. As we showed, bringing these scarce commodities into people’s homes sometimes generated expectations for medicines and care, especially in more deprived settings. Although our field teams did not feel unsafe in the field, they did encounter some difficult situations. In consultation with our field teams, we partially addressed the challenge by removing the use-by dates on the antibiotics and informing respondents that the antibiotics had expired. However, our teams still often felt compelled to help people access care in cases of dire need, such as driving them to the hospital or paying for transport or medicines. Social science scholarship emphasises the importance of ‘relational ethics’ when engaging such dilemmas [28–30], and thus in all of our surveys we have supported our field teams’ decision-making in the field, worked with them to overcome ethical issues, and held regular debriefings.

It is also of ethical and methodological significance that, by introducing people to antibiotics we had bought locally ‘over the counter’, the Drug Bag method may have demonstration effects on ABU practices. Were we sending people the ‘wrong message’ (e.g. that they should be using antibiotics)? During the consent process, we carefully explained that we had brought these antibiotics for research purposes to help respondents to remember any medicines that they have already encountered. We also explained that we were not medical/veterinary ‘experts’, nor there to judge or impose certain values, but rather social scientists interested in *their* opinions and experiences. From an anthropological perspective, it is unsurprising that the drug bags nonetheless generated new knowledge and practices: ethnographic research has long shown that global health research inevitably shapes the reality that it seeks to represent [31–33]. From a relational ethics perspective, we did not consider it problematic that some respondents, most living with limited access to medicines and/or care, gained knowledge that might have been useful for themselves and their families. However, this could well affect the reliability of ABU data, especially in the context of farming where we found the most examples of altered practices, and researchers seeking greater impartiality may seek ways of mitigating such effects.

Table 2 summarises the strengths and limitations of the method highlighted above.

**Table 2.** Observed strengths and limitations

	Strengths	Limitations
Data	Establishing patterns of recognition in settings with a relatively small and stable range of antibiotics Capturing ABU and approx. frequency of use Capturing symptoms and illnesses that freq. used antibiotics were used to treat	Unreliable data on recognition (and thus use/frequent use) in settings with wide and shifting range of antibiotics Directly identifying antibiotics that are difficult to access Demonstration effects on ABU practices
Logistical	Establishing relationships with providers for ongoing fieldwork Engaging and fun method, contributing to high recruitment rates	Time and resource intensive
Ethical	Takes seriously the importance of local categories, contexts and concerns	Generated expectations for medicines and care

### Considerations for larger surveys

In our anthropological surveys, the Drug Bag method was used primarily as a way of identifying themes and questions to be followed up ethnographically and was therefore more of a starting point than an end in and of itself. The potential to use this method to capture more widely generalisable ABU data rests on the following considerations. First, broadening the geographical scale requires compiling drug bag contents that are locally relevant but not too numerous. Compiling different bags according to locale may be required but raises challenges of comparability, unless the bags hold constant the same ingredients and modes of administration, while allowing considerable differences in brand and appearance. To avoid the problem of too many drugs in the bag, a smaller range of antibiotics could be included and the objectives narrowed to interest in particular types of medicines. Such targeted drug bags could also be more appropriate in settings where large, rapidly shifting markets for antibiotics makes for very large, unwieldy drug bags (as was the case in Myanmar). Given the centrality in this method of establishing recognition, data are likely to be more accurate if more/all versions of fewer antibiotic types are included than a few versions of more antibiotics.

Second, when designing survey contents, our experience points to the importance of including fewer, well-tested questions that can produce reliable data. Consideration for expanding this method should show caution in adding further questionnaire modules which are ill-suited to survey methodology – for example to capture phenomena such as ‘attitudes’, ‘beliefs’ and ‘culture’ [7,14,15]. To attempt this risks misleading ABU data and potentially reinforcing decontextualized imaginings of ‘irrational’ end users [27]. We recommend that understanding context is essential but that this requires other methods. Third, and relatedly, fieldworkers must feel empowered to report their experiences and challenges through the research process. Data interpretation relied on hearing about the how people engaged with the drug bags, which was made easier given the inbuilt qualitative component in most of our surveys. But in the context of larger surveys, qualitative data might not be collected, and thus it is all the more important to ensure feedback loops with researchers insulated from the ‘noise’ of the field. As discussed above in relation to ethics, field workers are a valuable and yet chronically undervalued resource in global health research, and their inclusion in data interpretation is crucial in this complex field of study. Finally, if this method were to be scaled up it would be valuable to compare it with other methodologies such as show cards, and consider whether the value of the data generated from the drug bag is worth the cost involved when carrying out a large-scale survey, or indeed whether there might be a role for the drug bag method earlier on in the design of tools for a larger scale survey.

## Conclusions

In this article, we presented findings and reflections from using the Drug Bag method to study ABU at the household and farm level in low-income settings. Overall, we found the method a useful way of commencing ethnographic research and have considered the wider applicability of the method for survey-based research. Further detailed comparison is needed to ascertain how and under what circumstances the method should be used instead of, or in conjunction with, other tools. Furthermore, careful thought is needed to decide how household ABU metrics should be collected and interpreted in relation to data collected at other levels, including provider and macro consumption data. We share these findings to add to the growing conversation about ABU data collection methods in spaces beyond prescriber settings where data are fewest and challenging to collect.

## Paper context

Valid and reliable community-level antibiotic use data are difficult to collect through established survey methods because of the limitations of respondent recall and translatability of the concept of ‘antibiotic’. The ‘Drug Bag’ method was designed to overcome these difficulties by presenting physical specimens of antibiotics to respondents. This paper presents experiences of using drug bags in Africa and South-East Asia.

## References

[1] World Health Organisation (WHO) Global action plan on antimicrobial resistance. Geneva: World Health Organisation; 2015 Available from: https://www.who.int/antimicrobial-resistance/global-action-plan/en/

[2] World Health Organisation (WHO) WHO report on surveillance of antibiotic consumption: 2016–2018 early implementation. Geneva: World Health Organisation; 2018 Available from: https://www.who.int/medicines/areas/rational_use/oms-amr-amc-report-2016-2018/en/

[3] IQVIA Institute for Human Data Science The global use of medicine in 2019 and outlook to 2023. New Jersey: IQVIA Institute; 2019 Available from: https://www.iqvia.com/institute/reports/the-global-use-of-medicine-in-2019-and-outlook-to-2023

[4] Versporten A, Zarb P, Caniaux I, et al Antimicrobial consumption and resistance in adult hospital inpatients in 53 countries: results of an internet-based global point prevalence survey. Lancet Glob Health. 2017;6:1–11. 10.1016/S2214-109X(18)30186-4 29681513

[5] Queenan K, Chandler CIR, Goodman C. A review of methods and metrics for studying human and livestock antibiotic use at the granular level: a pre-read for roundtable discussion in London, 21 & 22^nd^November 2017. 2017 Available from: https://amr.lshtm.ac.uk/wp-content/uploads/sites/12/2018/06/LSHTM-ABU-Metrics-and-Methods-Review-Nov17.pdf

[6] Das J, Hammer J, Sanchez-Paramo C. The impact of recall periods on reported morbidity and health seeking behaviour. J Dev Econ. 2012;98:76–88.

[7] Stone L, Campbell J The use and misuse of surveys in international development: an experiment from Nepal”. Hum Organ. 1984;43:27–37.

[8] Gikonyo C, Bejon P, Marsh V, et al Taking social relationships seriously: lessons learned from the informed consent practices of a vaccine trial on the Kenyan Coast. Soc Sci Med. 2008;67:708–720. 18362046 10.1016/j.socscimed.2008.02.003 PMC2682177

[9] Ribera JM, Hausmann-Muela S The straw that breaks the camel’s back: redirecting health-seeking behavior studies on malaria and vulnerability. Med Anthro Quarterly. 2011;25:103–121. 10.1111/j.1548-1387.2010.01139.x 21495497

[10] World Health Organisation (WHO) Antibiotic resistance: multi-country public awareness survey. Geneva: World Health Organization; 2015 Available from: http://apps.who.int/medicinedocs/documents/s22245en/s22245en.pdf

[11] Haenssgen M, Charoenboon N, Khine Zaw Y It is time to give social research a voice to tackle antimicrobial resistance? J Antimicrob Chemother. 2018;73:1112–1113. 29361004 10.1093/jac/dkx533

[12] Whyte SR, Van der Geest S, Hardon A Social lives of medicines. Cambridge: Cambridge University Press; 2002.

[13] Bernard HR Research methods in anthropology: qualitative and quantitative approaches. 5th ed. Lanham (Maryland): Altamira Press; 2011.

[14] Launiala A How much can a KAP survey tell us about people’s knowledge, attitudes and practices? Some observations from medical anthropology research on malaria in pregnancy in Malawi. Anthropol Matters. 2009;11 Available from: http://www.anthropologymatters.com/index.php/anth_matters/article/view/31/53

[15] Chandler CIR Knowledge, attitudes & practices surveys In: Callan H, editor. International encyclopedia of anthropology. London: Wiley; 2018.

[16] Sheridan J, Chamberlain K The power of things. Qual Res Psychol. 2011;8:315–332.

[17] Woodward S Object interviews, material imaginings and ‘unsettling’ methods: interdisciplinary approaches to understanding materials and material culture. Qual Res. 2016;16:359–374.

[18] Mayoux L, Chambers R Reversing the paradigm: quantification, participatory methods and pro-poor impact assessment. J Int Dev. 2005;17:271–298.

[19] Weller SC New data on intracultural variability: the hot-cold concept of medicine and illness. Hum Organiz. 1983;42:249–257.

[20] Hardon A Confronting ill health: medicines, self-care and the poor in Manilla. Quezon City (Philippines): Health Action Information Network; 1991.

[21] Trotter RT, Potter JM, Sorts P A cognitive anthropological model of drug and AIDS risks for Navajo teenagers: assessment of a new evaluation tool. Drugs Soc. 1993;7:23–39.

[22] Bourey C, Stephenson R, Bartel D, et al Pile sorting innovations: exploring gender norms, power and equity in sub-Saharan Africa. Glob Public Health. 2012;7:995–1008. 22866910 10.1080/17441692.2012.709259

[23] Haenssgen M, Charoenboon N, Althaus T, et al The social role of C-reactive protein point-of-care testing to guide antibiotic prescription in Northern Thailand”. Soc Sci Med. 2018;202:1–12. 29499522 10.1016/j.socscimed.2018.02.018 PMC5910303

[24] World Health Organisation (WHO) Model list of essential medicines. 20th list (March 2017). Geneva: World Health Organisation; 2017 Accessible from: https://www.who.int/medicines/publications/essentialmedicines/en/

[25] Matelski M On sensitivity and secrecy: how foreign researchers and their local contacts in Myanmar deal with risk under authoritarian rule. J Burma Stud. 2014;18:59–82.

[26] Metro R From the form to the face to face: iRBs, ethnographic researchers, and human subjects translate consent. Anthropol Educ Quart. 2014;45:167–184.

[27] Chandler CIR, Hutchinson E, Hutchison C Addressing antimicrobials through social theory: an anthropolgically oriented report. London: London School of Hygiene and Tropical Medicine; 2016 Available from: http://app.lshtm.ac.uk/files/2016/11/LSHTMAnthroAMR-2016.pdf

[28] Geissler PW, Kelly A, Imoukhuede B, et al “He is now like a brother, I can even give him some blood” Relational ethics and material exchanges in a malaria vaccine “trial community” in The Gambia. Soc Sci Med. 2008;67:696–707. 18455854 10.1016/j.socscimed.2008.02.004

[29] Molyneux S, Kamuya D, Madiega PA, et al Field workers at the interface. Dev World Bioeth. 2013;13:ii–iv. 10.1111/dewb.12027 PMC3662993 23521824

[30] Aellah G, Chantler T, Geissler PW Global health research in an unequal world: ethics case studies from Africa. Oxford: CAB International; 2016.

[31] Lock M, Nguyen V-K An anthropology of biomedicines. New Jersey: Wiley-Blackwell; 2010.

[32] Geissler PW, Molyneux S, eds. Evidence, ethos and experiment: the anthropology and history of medical research in Africa. New York: Berghahn Books; 2011.

[33] Biruk C Cooking data: culture and politics in an African research world. Durham: Duke University Press; 2018.

